# Tenascin‐c renders a proangiogenic phenotype in macrophage *via* annexin II

**DOI:** 10.1111/jcmm.13332

**Published:** 2017-08-30

**Authors:** Zhiyang Wang, Qi Wei, Liang Han, Keqing Cao, Tianfeng Lan, Zhenjie Xu, Yingjuan Wang, Yuan Gao, Jing Xue, Fei Shan, Jun Feng, Xin Xie

**Affiliations:** ^1^ Key Laboratory of Resource Biology and Biotechnology in Western China Ministry of Education College of Life Science Northwest University Xi'an China; ^2^ Institute of Integrated Medical Information Xi'an China; ^3^ Department of Traditional Chinese Medicine College of Life Science Northwest University Xi'an China; ^4^ Department of Cardiovascular Surgery Affiliated Hospital of Yan'an University Yan'an China; ^5^ Department of Vascular Surgery the First Affiliated Hospital of Xi'an JiaoTong University Xi'an China

**Keywords:** tenascin‐c, annexin II, atherosclerosis, macrophages, hypoxia

## Abstract

Tenascin‐c is an extracellular matrix glycoprotein, the expression of which relates to the progression of atherosclerosis, myocardial infarction and heart failure. Annexin II acts as a cell surface receptor of tenascin‐c. This study aimed to delineate the role of tenascin‐c and annexin II in macrophages presented in atherosclerotic plaque. Animal models with atherosclerotic lesions were established using ApoE‐KO mice fed with high‐cholesterol diet. The expression of tenascin‐c and annexin II in atherosclerotic lesions was determined by qRT‐PCR, Western blot and immunohistochemistry analysis. Raw 264.7 macrophages and human primary macrophages were exposed to 5, 10 and 15 μg/ml tenascin‐c for 12 hrs. Cell migration as well as the proangiogenic ability of macrophages was examined. Additionally, annexin II expression was delineated in raw 264.7 macrophages under normal condition (20% O_2_) for 12 hrs or hypoxic condition (1% O_2_) for 6–12 hrs. The expression of tenascin‐c and annexin II was markedly augmented in lesion aorta. Tenascin‐c positively regulated macrophage migration, which was dependent on the expression of annexin II in macrophages. VEGF release from macrophages and endothelial tube induction by macrophage were boosted by tenascin‐c and attenuated by annexin II blocking. Furthermore, tenascin‐c activated Akt/NF‐κB and ERK signalling through annexin II. Lastly, hypoxia conditioning remarkably facilitates annexin II expression in macrophages through hypoxia‐inducible factor (HIF)‐1α but not HIF‐2α. In conclusion, tenascin‐c promoted macrophage migration and VEGF expression through annexin II, the expression of which was modulated by HIF‐1α.

## Introduction

Monocytes in serum can respond to the accumulation of serum lipids and their derivants in some specific areas of the artery 1, 2, 3. Following the activation, monocytes then differentiate into macrophages and engulf modified lipoprotein, transforming into foam cells 2, 4. These cells along with other inflammatory mediators further aggravate the progression of atherosclerosis 5. Meanwhile, macrophages in atherosclerotic plaque are immersed in a microenvironment with multiple stimuli, such as interleukins, chemokines, oxidized LDL and extracellular matrix 6, 7, 8, 9. After the stimulation, the phenotypes of macrophages are altered and in turn affect atherosclerotic plaque stability.

Tenascin‐c is a hexameric extracellular glycoprotein, with four distinct domains: EGF‐L, FNIII, FBG and assembly domain 10. Tenascin‐c is highly expressed in the early embryo and is involved in the development process of the heart although deletion of its expression has no impact on normal development in mice 11, 12. However, its expression remains scarce in healthy adult heart tissue. Its expression will be triggered and re‐expressed when the heart is attacked by a variety of cardiac diseases or cardiac injury 13, 14. The expression of tenascin‐c has been a reliable biomarker of disease progression. Notably, tenascin‐c has been implicated in atherosclerosis progression. For example, tenascin‐c that expressed in advanced atherosclerotic plaque will recruit platelets *via* interacting with integrin α2β1 and induce platelet activation that is relevant to atherothrombosis 15. The level of tenascin‐c in atherosclerotic plaque is correlated with the infiltration of macrophages 16. Furthermore, tenascin‐c can act as damage‐associated molecular patterns (DAMPs) to activate macrophages and fibroblasts and up‐regulate inflammatory cytokine expression through TLR‐4 receptor 17, 18.

Annexin II is a pleiotropic protein that can bind anionic phospholipids in a calcium‐dependent manner 19. This protein is involved in diverse cellular processes, such as cell motility, endocytosis, fibrinolysis, ion channel formation and cell matrix interactions 20. Recently, several reports have characterized the role of annexin II in innate immunity and inflammatory response. Knockdown of annexin II expression reduced the phagocytic ability of macrophages, whereas treating with extracellular recombinant protein promoted phagocytosis 21. Annexin II also plays a role in TLR4 internalization and translocation into endosome, which contributes to anti‐inflammatory/pro‐inflammatory cytokines release from macrophages 22. Furthermore, the expression of annexin II was increased in vascular neointima and mediated vascular smooth muscle cell migration and proliferation, facilitating atherosclerotic plaque formation 23. Of note, annexin II is a cell surface receptor of tenascin‐c. Their interaction leads to the alteration of cell growth, migration, adhesion and chemoresistance 24. Additionally, annexin II receptor is involved in the activation of downstream NF‐κB, ERK1/2, mTOR and PI3K signalling pathway, suggesting a multifaceted role for annexin II in human health and disease 25, 26, 27.

In the present study, we sought to examine the expression of tenascin‐c and annexin II in the atherosclerotic plaque of apoE‐KO mice and explore the effect of the interaction between tenascin‐c and annexin II on macrophage behaviour *in vitro*. Our research may provide novel insight into the mechanism of macrophages implicated in atherosclerosis progression.

## Materials and methods

### Animals

To examine the expression of tenascin‐c and annexin II, ApoE‐KO mice of 5‐week old from a C57BL/6J background were chosen. ApoE‐KO male and female mice were provided by Shanghai Laboratory Animal Center (Shanghai, China). To induce atherosclerotic lesions, the mice were fed an intensively high‐cholesterol diet (0.2% weight cholesterol; 15.2% kcal protein, 42.7% kcal carbohydrate, 42.0% kcal fat) for 16 weeks. All animal care and procedures were reviewed and approved by the Ethics Committee of Northwest University.

### Immunohistochemistry analysis

The expression of tenascin‐c and annexin II protein in mice aorta was analysed by immunohistochemical staining 28. Briefly, arterial sections (5 μm thick) were fixed in 5% formaldehyde before dehydrated with different doses of ethanol. The sections were then adhered to poly‐l‐lysine glass slides, washed with phosphate‐buffered saline (PBS 0.01 M, pH 7.4) and then treated with hydrogen peroxide to inactivate endogenous peroxides. The samples were then blocked with 1% bovine serum albumin and incubated with anti‐tenascin‐c (1:200; Santa Cruz Biotech, Dallas, TX, USA) and anti‐annexin II (1:200; Santa Cruz Biotech) antibody for 12 hrs at 44°C. After washing with PBS for three times, the sections were incubated with secondary antibody for 10 min. at 37°C and then incubated with horseradish peroxidase labelled streptavidin solution. Diaminobenzidine (DAB) was used for stain, with incubation for 1–2 min. The cover slips were counterstained with haematoxylin and then examined under light microscopy.

### Cell culture and treatments

In all, 20 healthy volunteers aged between 30 and 50 years were recruited and the blood samples were obtained. Ficoll Paque (GE Healthcare, Freiburg, Germany) density gradient centrifugation was then employed to prepare peripheral blood mononuclear cells (PBMCs). Human monocytes were isolated from PBMCs by the monocyte isolation kit (Miltenyi Biotec, Shanghai, China) and then cultured in RPMI medium supplemented with 10% foetal bovine serum (FBS) at 37°C in 5% CO_2_. Monocytes were then stimulated with 50 ng/ml granulocyte macrophage colony‐stimulating factor (GM‐CSF) for 7 days to differentiate into macrophages. Raw 264.7 macrophages (ATCC, Manassas, VA, USA) were cultured in DMEM/High Glucose (Thermo Fisher, Shanghai, China) supplemented with 10% cosmic calf serum (GE Healthcare). Cells were maintained at 37°C in an atmosphere of 5% CO_2_. Hypoxic condition was created using a hypoxic work station with 1% O_2_, 94% N_2_ and 5% CO_2_ at 37°C. The procedure on human cell line was performed in accordance with the Declaration of Helsinki. Our experiments have been approved and reviewed by the Ethics Committee of Northwest University.

For tenascin‐c exposure, human primary macrophages and raw 264.7 macrophages were exposed to 5, 10 and 15 μg/ml tenascin‐c (purity: >97% pure by SDS‐PAGE, EMD Millipore, Billerica, MA, USA) for 12 hrs. After the treatment, macrophages, as well as their culture supernatants, were collected for analysis. For annexin II blocking, macrophages were treated with IgG or 2 μg/ml anti‐annexin II antibody for 2 hrs. For LY294002 (PI3K inhibitor) and U0126 (ERK1/2 inhibitor) treatment, raw 264.7 macrophages were pre‐treated with 10 μM LY294002, or 10 μM U0126 for 30 min. followed by 15 μg/ml tenascin‐c for 12 hrs. After the treatment, culture supernatants were removed and raw 264.7 macrophages were collected for further analysis.

### Macrophage migration assay

Migration assays were assessed by transwell cell culture chambers 5 mm pores (Corning Incorporated, Corning, NY, USA). In brief, 1 × 10^5^ pre‐treated macrophages were suspended in 100 ml DMEM containing 0.5% FBS and added to the upper compartment of the insert. The lower chamber was filled with 100 ml DMEM containing 10% FBS. After incubation for 6 hrs at 37°C in a 5% CO_2_ incubator, cells were scraped from the upper surface, duplicate membranes fixed and migrated cells stained with crystal violet. Cells were counted in five random fields.

### Annexin II siRNA and pcDNA3.1‐annexin II transfection

Scramble small interfering RNA (siRNA, sc‐37007) and annexin II siRNA (sc‐29683) were provided by Santa Cruz Biotech Inc. To construct annexin II overexpression vector, the full length of the annexin II gene was cloned and then inserted into EcoRI/BamHI sites of a pcDNA3.1 plasmid. For siRNA/plasmid transfection, raw 264.7 macrophages were dissociated into single cells in suspension and plated on six‐well plates (1 × 10^5^ cells/well). Cells were transfected with annexin II siRNA/pcDNA3.1‐annexin II according to the manufacturer's protocol. Cells were harvested at the indicated time for further analysis. The efficiency of the annexin II siRNA/pcDNA3.1‐annexin II transfection was confirmed by Western blot analysis.

### Detection of VEGF release

Human primary macrophages or raw 264.7 macrophages were exposed to 0–15 μg/ml tenascin‐c for 12 hrs. After this exposure, the supernatants of the cell cultures were collected and presented for the determination of VEGF release by ELISA kits (R&D Systems, Minneapolis, MN, USA). The expression was normalized to the cell counts (1 × 10^5^ cells/well).

### Co‐culture of raw 264.7 macrophage and endothelial cells

For the co‐culture of raw 264.7 macrophages and endothelial cells, ice‐cold Matrigel was placed into 24‐well plates and polymerized for 30 min. at 37°C. Human umbilical vein endothelial cells (HUVECs; Lonza Group Ltd, Basel, Switzerland) were starved for 24 hrs in DMEM with 5% FBS and seeded onto the Matrigel (5 × 10^4^ cells/well). Following the attachment, transwell chambers (Corning) with raw 264.7 macrophages (1 × 10^5^ cells/well, pre‐treated with or without 15 μg/ml tenascin‐c) were placed on the HUVECs. After 12 hrs, transwell chambers were removed and endothelial tube formation was observed. Five different fields were chosen randomly in each well and photographs were taken with a phase‐contrast microscope. The length of the tubes was measured using Image Pro Plus software(Media Cybernetics, Silver Spring, MA, USA) and expressed as the average length (micrometer) per microscopic field.

### qRT‐PCR

Total RNA was extracted from human primary macrophages or raw 264.7 macrophages after tenascin‐c exposure using Trizol Reagent according to the manufacturer's instructions (Biostar, Shanghai, China). About 5 μg total RNA for each sample was reverse‐transcribed into first strand cDNA for qRT‐PCR analysis. The qRT‐PCR was performed at a final volume of 10 μl which contained 5 μl of FastFire qPCR PreMix (Tiangen, Beijing, China), 1 μl of cDNA (1:50 dilution) and 2 μl each of the forward and reverse primers (1 mM). The following qRT‐PCR steps were performed: 94°C for 2 min. for the initial denaturation; 94°C for 20 sec., 58°C for 15 sec. and 72°C for 15 sec., with 2 sec. for plate reading for 40 cycles; and a melt curve from 65 to 95°C. Β‐actin was used to normalize the gene expression. Detailed primer sequences used are listed in Table 1.

**Table 1:** Primer sequence used in qRT‐PCR analysis

Gene symbols	Sequence
Tenascin‐c	Forward: 5′‐GACCTGCCCTTTGCTGAC‐3′
	Reverse: 5′‐TTCCGTTCTTCTTGACTTCTG‐3′
Annexin II	Forward: 5′‐TGCCAGCGACATAACAGT‐3′
	Reverse: 5′‐CCAGATTCCCTCCAAAGC‐3′
VEGF	Forward: 5′‐CATTGGAGCCTTGCCTTG‐3′
	Reverse: 5′‐TTCGTGGGGTTTCTGGTCT‐3′
β‐actin	Forward: 5′‐GTGGACATCCGCAAAGAC‐3′
	Reverse: 5′‐TGGAAGGTGGACAGCGAGGC‐3′

### Western blot

Lysates of aorta or macrophages were prepared and then separated by 10% SDS‐PAGE. Briefly, cells were homogenized and lysed with RIPA lysis buffer. 40 μg protein per lane was separated by 12% SDS–PAGE and electroblotted onto a poly (vinylidene difluoride) membrane (GE Healthcare). Following that, non‐specific binding was blocked by incubating with 5% non‐fat milk in TBST buffer at room temperature for 1 hr. The transferred proteins were incubated overnight at 4°C with various primary antibodies in Tris‐buffered saline with Tween buffer (20 mM Tris‐HCl, 150 mM NaCl and 0.1% Tween 20) followed by incubation with horseradish peroxidase‐conjugated secondary antibodies for 1 hr. Primary antibodies including anti‐tenascin‐c (1:500; Santa Cruz Biotech), anti‐annexin II (1:500; Santa Cruz Biotech), anti‐Akt (1:500; Santa Cruz Biotech), anti‐p‐Akt (1:500; Santa Cruz Biotech), anti‐p65 (1:500; Santa Cruz Biotech), anti‐p‐p65 (1:600; Santa Cruz Biotech), anti‐ERK1/2 (1:800; Santa Cruz Biotech), anti‐p‐ERK1/2 (1:800; Santa Cruz Biotech), anti‐HIF‐1α (1:500; Santa Cruz Biotech) and anti‐HIF‐2α (1:600; Abcam, Cambridge, MA, USA) were employed. After extensive washes in Tris‐buffered saline with Tween buffer, the signals on the membrane were visualized by enhanced chemiluminescence (GE Healthcare).

### Statistical analysis

All data were presented as Mean ± S.D. Statistical analysis was carried out using SPSS 17.0 software (SPSS Inc., Chicago, IL, USA). For difference comparisons of three or more groups, one‐way anova analysis followed by LSD *post‐hoc* tests was performed. Differences were considered to be significant when *P* < 0.05.

## Results

### Tenascin‐c and annexin II are highly expressed in atherosclerotic plaque

**Figure 1:**
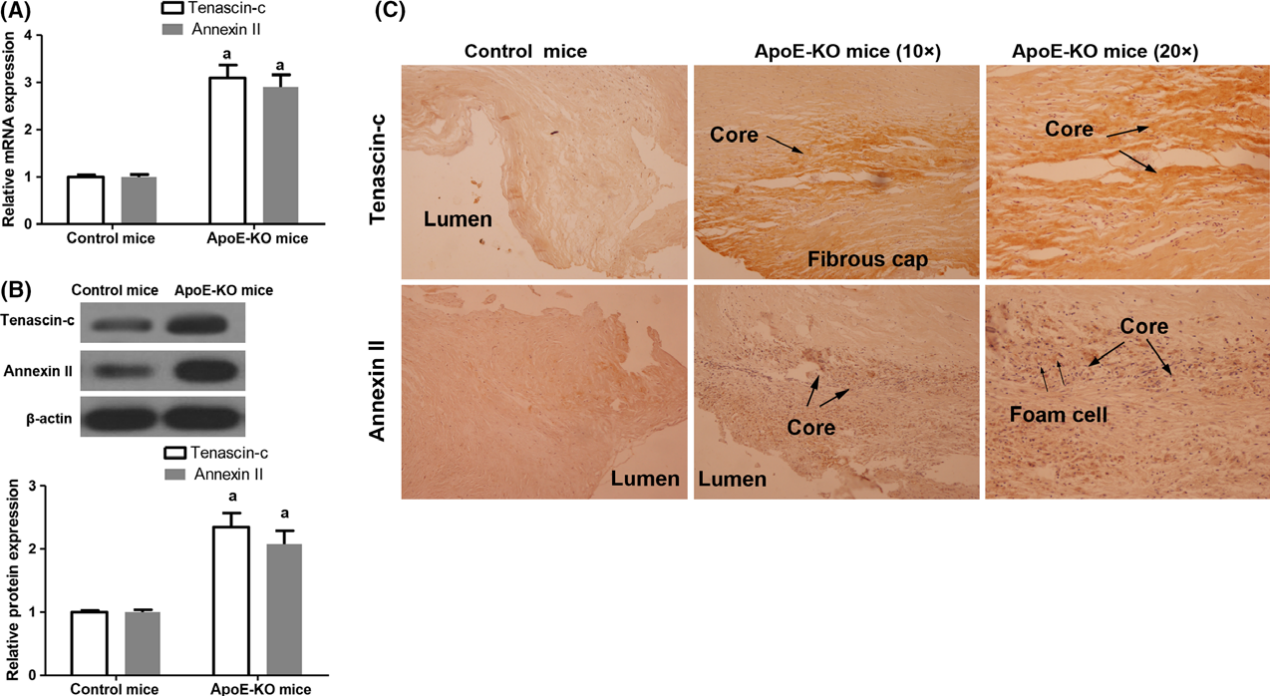
Examination of tenascin‐c expression and annexin II expression in aorta of normal mice or ApoE‐KO mice with atherosclerotic lesions. (**A**) Analysis of tenascin‐c mRNA and annexin II mRNA expression. Tenascin‐c: *n* = 6, *t* = 14.014, *P* < 0.001; annexin II:* n* = 6, *t* = 11.277, *P* < 0.001. (**B**) Analysis of tenascin‐c protein and annexin II protein expression by Western blot. Tenascin‐c: *n* = 6, *t* = 12.849, *P* < 0.001; annexin II:* n* = 6, *t* = 12.204, *P* < 0.001. (**C**) Evaluation of tenascin‐c protein and annexin II protein expression using immunohistochemical staining (left lane and middle lane 10×, right lane 20×). ^a^ *P* < 0.05 *versus* control mice.

To compare the expression of tenascin‐c and annexin II in atherosclerotic plaque, apoE‐KO and B6 control mice were fed with the standard atherogenic diet for 16 weeks to establish models with atherosclerotic lesions. After that, the aorta of these animals was isolated and the expression of tenascin‐c and annexin II was determined by qRT‐PCR. The results showed that compared to B6 control mice, the expression of tenascin‐c and annexin II mRNA was significantly elevated (Fig. 1A). Furthermore, protein expression of these two genes was also analysed by Western blot. The expression of tenascin‐c and annexin II protein was up‐regulated (Fig. 1B). Additionally, their expression was observed using immunohistochemical staining. We found that tenascin‐c was mostly expressed around the lipid core, whereas annexin II was expressed on endothelial cells, macrophages and foam cells (Fig. 1C). Therefore, our data revealed that both tenascin‐c and annexin II were highly expressed in atherosclerotic plaque.

### Annexin II plays a role in macrophage migration induced by tenascin‐c

**Figure 2:**
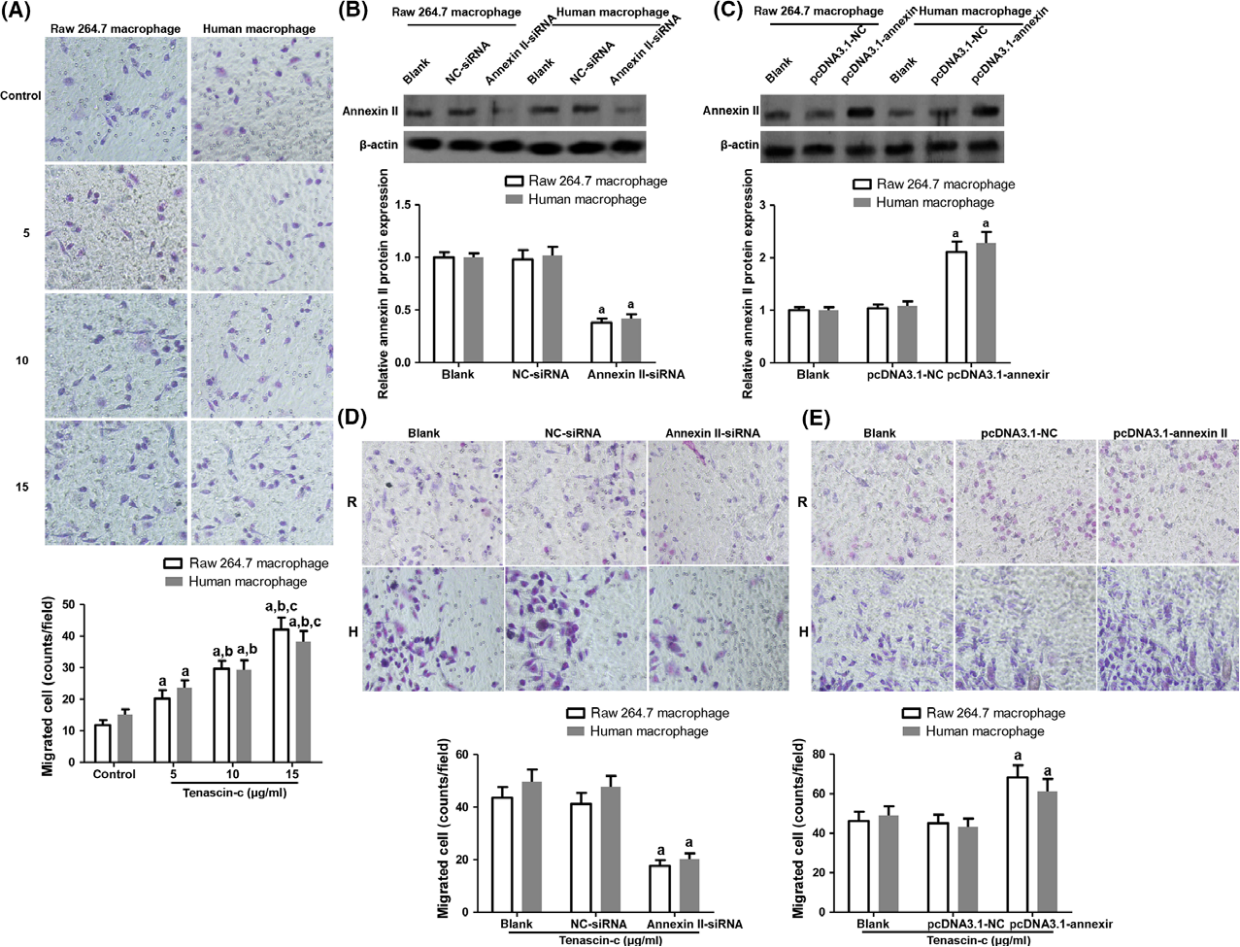
Induction of macrophage migration by tenascin‐c through annexin II. (**A**) Raw 264.7 macrophages and human primary macrophages were challenged with 5, 10 and 15 μg/ml tenascin‐c for 12 hrs and cell migration was evaluated by Transwell assay. Raw 264.7 macrophages: *n* = 8, *F* = 46.839, *P* < 0.001; primary macrophages: *n* = 8, *F* = 42.765, *P* < 0.001. (**B**) Annexin II expression was inhibited using annexin II siRNA. Raw 264.7 macrophages: *n* = 4, *F* = 18.027, *P* = 0.023; primary macrophages: *n* = 4, *F* = 15.685, *P* = 0.024. (**C**) Annexin II expression was enhanced by pcDNA3.1‐annexin II plasmid transfection. Raw 264.7 macrophages: *n* = 4, *F* = 21.353, *P* = 0.018; primary macrophages: *n* = 4, *F* = 22.714, *P* = 0.017. (**D**) and (**E**) Macrophage migration was detected when annexin II expression was knocked down/promoted. R indicates Raw 264.7 macrophages; H indicates human primary macrophages; For siRNA transfection: Raw 264.7 macrophages: *n* = 8, *F* = 35.297, *P* < 0.001; primary macrophages: *n* = 8, *F* = 36.683, *P* < 0.001. For plasmid transfection, Raw 264.7 macrophages: *n* = 8, *F* = 13.282, *P* = 0.003; primary macrophages: *n* = 8, *F* = 12.269, *P* = 0.004. ^a^ *P* < 0.05 *versus* control or blank group; ^b^ *P* < 0.05 *versus* 5 μg/ml tenascin‐c group; ^c^ *P* < 0.05 *versus* 10 μg/ml tenascin‐c.

Previous studies have reported that tenascin‐c may directly interact with annexin II and exert several regulatory functions 29. Annexin II was expressed on macrophages, the major inflammatory cells that play critical parts in atherosclerotic plaque formation 1, we took up to investigate the role of annexin II in macrophages with tenascin‐c circumstance. Raw 264.7 macrophages and human primary macrophages were challenged with 5, 10 and 15 μg/ml tenascin‐c for 12 hrs and cell migration was evaluated by Transwell assay. Compared to the control, macrophage migration was promoted by tenascin‐c in a dose‐dependent manner (Fig. 2A). Next, annexin II expression in macrophages was silenced using annexin II siRNA or overexpressed by pcDNA3.1‐annexin II transfection (Fig. 2B and C). The data showed that knockdown of annexin II impeded macrophage migration, whereas overexpression of annexin II promoted cell migration (Fig. 2D and E). These results suggested that annexin II is important for macrophage migration induced by tenascin‐c.

### Annexin II contributes to increased VEGF expression in macrophage

**Figure 3:**
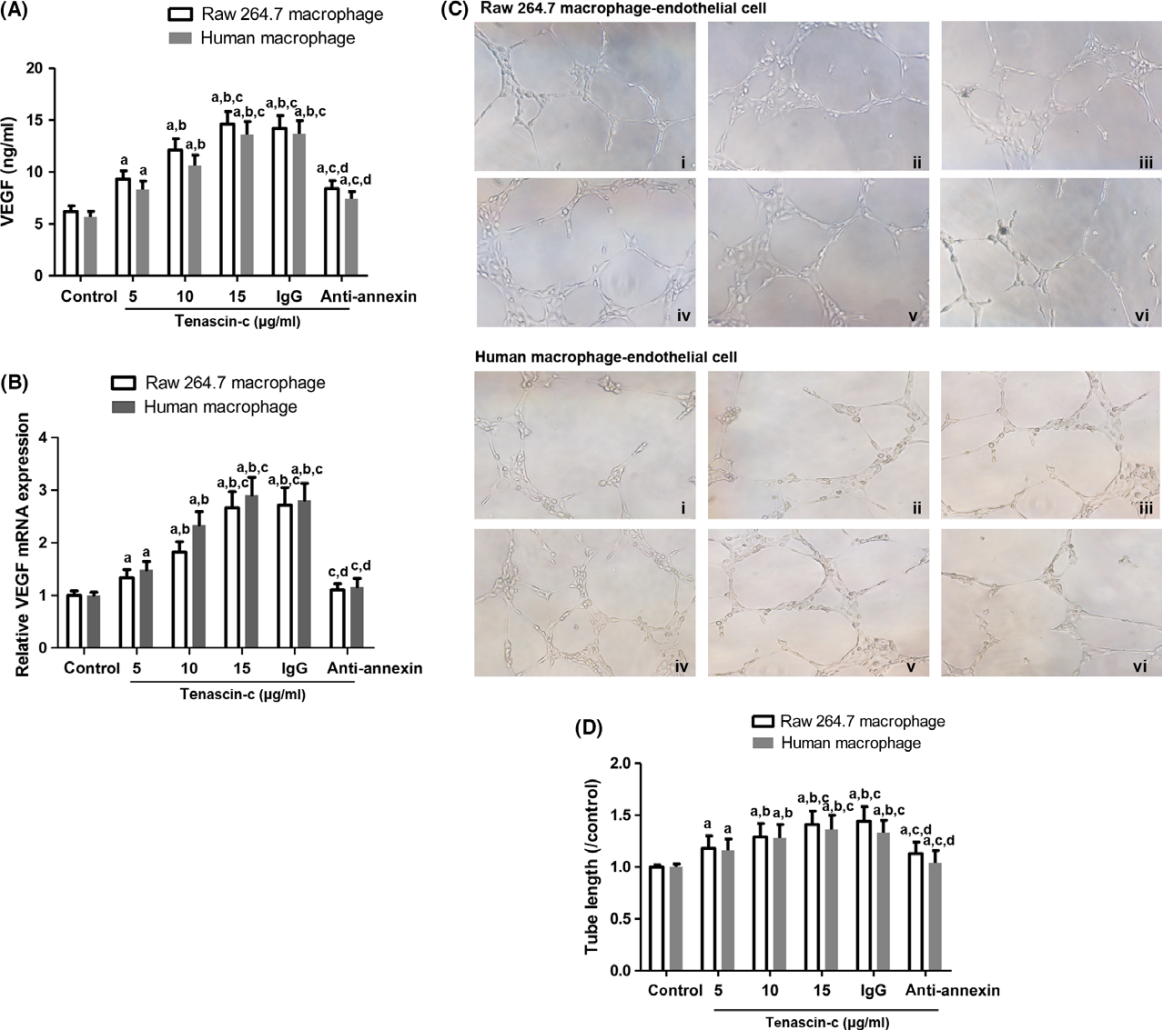
Effect of tenascin‐c on macrophage proangiogenic ability. (**A**) VEGF release from macrophages after tenascin‐c exposure (5, 10 and 15 μg/ml, 12 hrs). Raw 264.7 macrophages: *n* = 6, *F* = 29.453, *P* = 0.001; primary macrophages: *n* = 6, *F* = 24.706, *P* = 0.003. (**B**) VEGF mRNA expression was evaluated by qRT‐PCR. Raw 264.7 macrophages: *n* = 4, *F* = 28.526, *P* = 0.011; primary macrophages: *n* = 4, *F* = 26.119, *P* = 0.012. (**C**) Endothelial tube formation by endothelial cells after co‐cultured with macrophages. Macrophages were pre‐treated with i. control group; ii. 5 μg/ml tenascin‐c; iii. 10 μg/ml tenascin‐c; iv. 15 μg/ml tenascin‐c; v. IgG+15 μg/ml tenascin‐c; vi. anti‐annexin II antibody+15 μg/ml tenascin‐c. (**D**) Calculation of tube length. Raw 264.7 macrophages: *n* = 8, *F* = 11.069, *P* = 0.004; primary macrophages: *n* = 8, *F* = 9.177, *P* = 0.006. ^a^ *P* < 0.05 *versus* control; ^b^ *P* < 0.05 *versus* 5 μg/ml tenascin‐c group; ^c^ *P* < 0.05 *versus* 10 μg/ml tenascin‐c; ^d^ *P* < 0.05 *versus* IgG group.

Tenascin‐C has been involved in angiogenesis as an extracellular signal molecule 30. Angiogenesis inside the arterial wall and atherosclerotic plaques contributes mostly to plaque vulnerability 31. Therefore, we examined whether tenascin‐c could confer a proangiogenic effect to macrophages. Raw 264.7 macrophages and human primary macrophages were challenged with 5, 10 and 15 μg/ml tenascin‐c for 12 hrs. After that, VEGF secretion in the supernatant was determined by ELISA assay. The data revealed that tenascin‐c markedly prompted VEGF secretion and mRNA expression in macrophages (Fig. 3A and B). However, when annexin II in macrophages was blocked by anti‐annexin II antibody, VEGF secretion and mRNA expression were impeded in those cells (Fig. 3A and B). Likewise, the effect of macrophage on endothelial tube formation was also evaluated. Compared with the control, when macrophages were pre‐treated with tenascin‐c, endothelial tube formation was significantly boosted, which was attenuated by annexin II blocking (Fig. 3C and D). Our results indicated that annexin II is important for tenascin‐c‐induced VEGF expression and endothelial tube formation.

### Annexin II is responsible for Akt/NF‐κB and ERK activation induced by tenascin‐c

**Figure 4:**
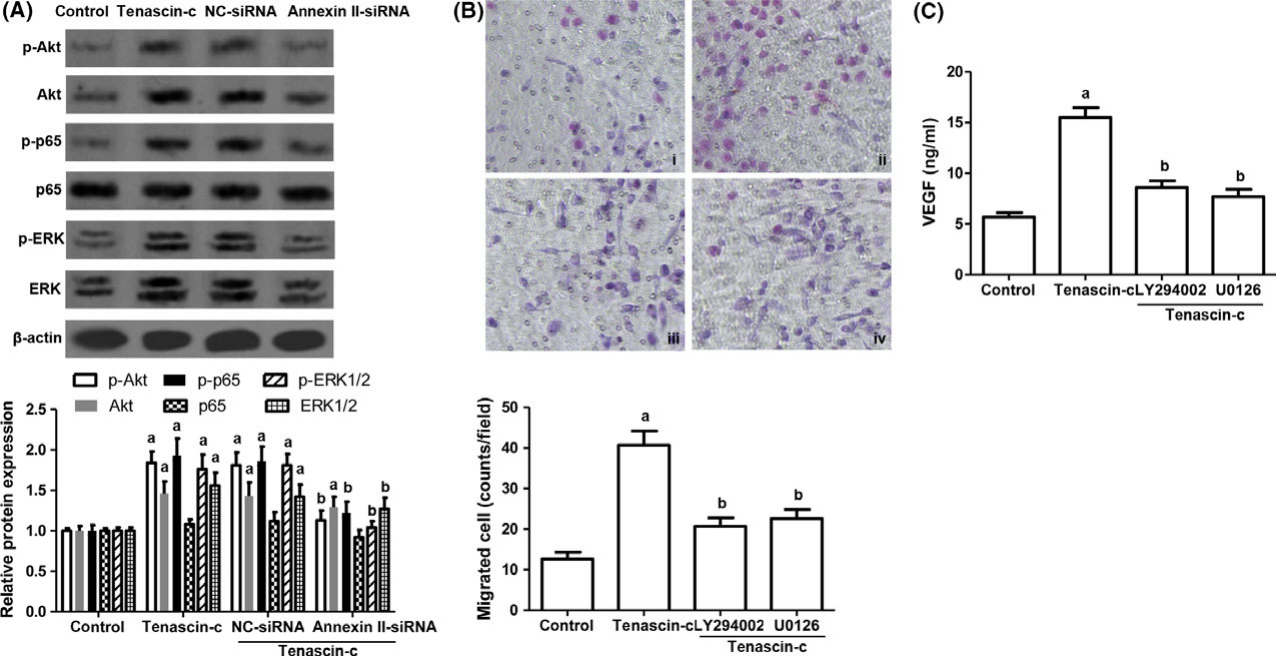
Regulation of Akt/NF‐κB and ERK signalling pathway by tenascin‐c. (**A**) The expression of p‐Akt, Akt, p‐p65, p65, p‐ERK1/2, ERK1/2 in raw 264.7 macrophages with tenascin‐c treatment and annexin II knockdown. NC‐siRNA indicates scramble small interfering RNA (siRNA) transfection group; annexin II‐siRNA indicates annexin II siRNA transfection group. p‐Akt: *n* = 4, *F* = 17.097, *P* = 0.024; Akt: *n* = 4, *F* = 13.206, *P* = 0.029; p‐p65: *n* = 4, *F* = 18.229, *P* = 0.023; p‐ERK1/2: *n* = 4, *F* = 18.142, *P* = 0.023; ERK1/2: *n* = 4, *F* = 15.395, *P* = 0.025. (**B**) Observation of raw 264.7 macrophage migration pre‐treated with LY294002 (10 μM, PI3K inhibitor) and U0126 (10 μM, ERK1/2 inhibitor). i. control group; ii. tenascin‐c group; iii. LY294002 + tenascin‐c group; iv. U0126 + tenascin‐c. *n* = 8, *F* = 30.282, *P* < 0.001. (**C**) VEGF release from raw 264.7 macrophages with LY294002 and U0126 pre‐conditioning. *n* = 6, *F* = 26.895, *P* = 0.002. ^a^ *P* < 0.05 *versus* control; ^b^ *P* < 0.05 *versus* tenascin‐c group.

In the following, we further explored the mechanism of annexin II involved in macrophage activation through examining several signal molecules including Akt, NF‐κB and ERK1/2. Compared with the control, the relative expression of phosphorylated Akt (p‐Akt), p‐p65 and p‐ERK1/2 in macrophages was stimulated by tenascin‐c exposure (Fig. 4A). Nonetheless, when annexin II expression was inhibited by annexin siRNA, the expression of p‐Akt, p‐p65 and p‐ERK1/2 was dampened in macrophages (Fig. 4A). Furthermore, raw 264.7 macrophages were pre‐treated with LY294002 (PI3K inhibitor) and U0126 (ERK1/2 inhibitor) before tenascin‐c exposure. These inhibitors markedly regressed macrophage migration and VEGF expression induced by tenascin‐c (Fig. 4B and C). The results revealed that annexin II mediated Akt/NF‐κB and ERK signalling pathway activation in macrophages induced by tenascin‐c.

### Hypoxia conditioning facilitates annexin II expression in macrophages

**Figure 5:**
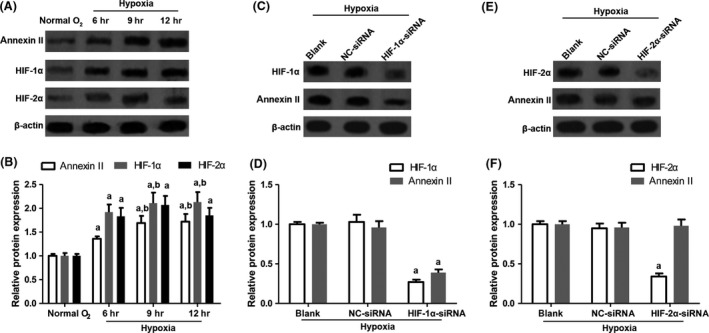
Effect of hypoxia on the expression of annexin II, HIF‐1α and HIF‐2α. (**A**,** B**) Raw 264.7 macrophages were exposed to normal condition (20% O_2_) for 12 hrs or to hypoxic condition (1% O_2_) for 6–12 hrs. Then, the expression of annexin II, HIF‐1α and HIF‐2α was examined by Western blot. Annexin II:* n* = 4, *F* = 11.284, *P* = 0.038; HIF‐1α: *n* = 4, *F* = 21.338, *P* = 0.018; HIF‐2α: *n* = 4, *F* = 17.682, *P* = 0.024. (**C**) and (**D**) The expression of annexin II in macrophages with HIF‐1α knockdown. HIF‐1α: *n* = 4, *F* = 23.325, *P* = 0.015; annexin II:* n* = 4, *F* = 21.893, *P* = 0.018. (**E**,** F**) The expression of annexin II in macrophages with HIF‐2α knockdown. HIF‐2α: *n* = 4, *F* = 26.314, *P* = 0.012; annexin II:* n* = 4, *F* = 0.894, *P* = 0.559. ^a^ *P* < 0.05 *versus* normal O_2_ group or blank group; ^b^ *P* < 0.05 *versus* 6 hrs group.

Additionally, because hypoxia is commonly found in atherosclerotic plaques in humans and animal models and promotes atherosclerosis 32, we sought to explore whether hypoxia might regulate annexin II expression on macrophages. Raw 264.7 macrophages were exposed to normal condition (20% O_2_) for 12 hrs or to hypoxic condition (1% O_2_) for 6–12 hrs. Following that, annexin II expression was analysed. The results showed that annexin II protein was up‐regulated by hypoxia (Fig. 5A). At the same time, the expression of hypoxia‐inducible factor 1α (HIF‐1α) and HIF‐2α was also augmented by hypoxia (Fig. 5A and B). Interestingly, siRNA targeting HIF‐1α but not HIF‐2α significantly decreased annexin II expression in macrophages under hypoxia (Fig. 5C–F). Therefore, our results demonstrated that hypoxia might impact the expression of annexin II on macrophages *via* HIF‐1α.

## Discussion

Recently, emerging evidence has shown that plasma tenascin‐c significantly correlated with the characteristics of atherosclerotic plaques. In patients with ACS, tenascin‐c levels were increased in the ruptured group than in the stable angina pectoris (SAP) group 33. However, no significant difference was observed between the non‐ruptured ACS and SAP groups 33. Furthermore, in recent stroke patients, the levels of plasma tenascin‐c were found to be higher than those of controls 34. Tenascin expression was also significantly enhanced in the vascular tissues with the plaque morphology of thrombus, angiogenesis and intraplaque haemorrhage 35. Carotid artery plaques could be stabilized by the inhibition of pro‐inflammatory factors and tenascin‐c suppression 36. Annexin II is a type of cell surface receptor which can specifically interact with tenascin‐c 29. This receptor is involved in multiple processes such as exocytosis and endocytosis, cell cycle regulation and immunoglobulin transport 37. Additionally, annexin II also mediates endothelial cell activation and plays a critical role in the process of thrombosis for anti‐phospholipid syndrome 38.

In our observation, the expression of tenascin‐c and annexin II was elevated in atherosclerotic plaques of a mouse model. Specifically, tenascin‐c expression focused around the lipid core and annexin II was mainly expressed on endothelial cells, macrophages and foam cells (Fig. 1). In contrast, some previous studies on mice with tenascin‐c deletion revealed a protective role for tenascin‐c in atherosclerosis and showed that tenascin‐c signalling might have the potential to reduce atherosclerosis 39, 40. As atherosclerosis progression is a complex regulatory process which comprises multiple vascular components and immune responses, these controversial results may attribute the different roles of mediators involved in the formation of atherosclerotic plaques.

In this study, transwell assay results showed that macrophage migration was prompted by tenascin‐c exposure. And annexin II was demonstrated to mediate the effect of tenascin‐c on macrophage migration (Fig. 2). Furthermore, VEGF secreted by macrophage was also up‐regulated by tenascin‐c through interacting with annexin II. VEGF modulation led to a variation of tube formation in macrophage‐endothelial cell co‐culture system (Fig. 3). These results indicated the proangiogenic phenotype induced by tenascin‐c. Of note, augmented expression of VEGF is a stimulator for intraplaque neovascularization, which may result in intraplaque haemorrhages and unstable plaque transition. Adhesion of monocytes to endothelial cells of lesion‐prone sites in arteries is mediated by cell adhesion molecules including VCAM‐1, P‐selectin, E‐selectin, ICAM‐1, etc. 41. Besides, MCP‐1, CCR‐2 and oxidized LDL drive monocyte migration and differentiation into macrophages and form foam cells 41. Evidence of tenascin‐c in regulation of cell adhesion molecules is scarce although deletion of tenascin‐c even sensitizes the reaction of VCAM‐1 to TNF‐α stimulation 39.

Annexin II mediated activation of Akt signalling and ERK1/2 signalling is involved in plasminogen activator induced cell proliferation 27. Furthermore, annexin II expression in macrophage contributes to Akt/NF‐κB and ERK1/2 activation induced by plasmin and resulted in cytokine expression 42. Our results suggested that tenascin‐c could seduce the activation of Akt/NF‐κB and ERK1/2 *via* annexin II. Inactivating Akt/NF‐κB and ERK1/2 pathway using inhibitors such as LY294002 and U0126 would alleviate VEGF expression and migration of macrophages under tenascin‐c conditioning (Fig. 4), suggesting Akt/NF‐κB and ERK1/2 pathway are involved in tenascin‐c induced effect on macrophages. The doses of LY294002 and U0126 have been validated in either macrophage cell lines or human macrophages in several previous studies 43, 44. Activation of Akt/NF‐κB and ERK1/2 has been shown to enhance pro‐inflammatory cytokine production and matrix metalloproteinases (MMPs) expression in macrophages 45, 46. Therefore, it is possible that tenascin‐c would drive macrophage migration and recruitment through the augmented MMPs and cytokines.

The supply of oxygen to the atherosclerotic plaque core is limited in symptomatic patients 47, 48. Hypoxic condition was also demonstrated in animal models and had an impact on macrophage lipid metabolism 49. In our study, we found that hypoxia treatment could enhance annexin II expression, with increasing HIF‐1α and HIF‐2α. When the expression of HIF‐1α was inhibited, annexin II expression was decreased (Fig. 5). Thus, our results revealed that hypoxia can modulate annexin II expression in macrophages through HIF‐1α. Our results were in line with Huang *et al*.^50^ study. P53 is another critical regulator that involved in the pathogenesis of atherosclerosis. Loss of p53 accelerates atherosclerosis development and plaque remodelling 51. P53 is also a regulator for annexin II expression 52. Therefore, in the early stage of atherosclerosis, p53 inactivation may be responsible for annexin II accumulation in macrophages infiltrated in aortic lesions.

In conclusion, our study demonstrated an increasing expression of tenascin‐c and annexin II in atherosclerotic plaque. The interaction between tenascin‐c and annexin II facilitated macrophage migration and VEGF expression *via* Akt, NF‐κB and ERK1/2 pathway. A solution targeting their interaction might help to reduce macrophage infiltration and angiogenesis in atherosclerosis. Nevertheless, a limitation of our research lies in the origin of tenascin‐c expression, which may emphasize our further research direction.

## Conflict of interest

The authors declare that there is no conflict of interest.
